# Commentary: A cross-continental comparative analysis of the neurological manifestations of Long COVID

**DOI:** 10.3389/fnhum.2026.1798243

**Published:** 2026-03-31

**Authors:** Luis Mesquita da Fonseca

**Affiliations:** AEEON, Esmoriz, Portugal

**Keywords:** cross-cultural research, global health, neuro-phenomenology, PASC, selection bias

## 1 Introduction

The study by Jimenez et al. (2026) provides a pioneering cross-continental analysis of the neurological sequelae following SARS-CoV-2 infection, encompassing a cohort of 3,100 patients across four countries. While the authors' effort to map the global burden of post-acute sequelae of COVID-19 (PASC) is commendable, the interpretation of the observed disparities—particularly the significantly lower prevalence of cognitive symptoms in Low-and-Middle-Income Countries (LMICs) compared to High-Income Countries (HICs)—warrants critical methodological scrutiny. This commentary addresses concerns regarding selection bias and the cultural invariance of neuro-phenomenological assessment tools.

## 2 Selection bias and recruitment disparities

A primary concern lies in the heterogeneity of the recruitment sources. As detailed in the original article, the HIC cohorts (USA) were predominantly derived from a specialized Neuro-COVID clinic, a setting that inherently attracts patients with high symptomatic self-awareness and localized neurological complaints. Conversely, the LMIC cohorts (e.g., India and Nigeria) were recruited through general post-acute follow-up programs or hospital settings.

Comparing a self-selected population seeking sub-specialized neurological care with a general post-infection population introduces a critical selection bias (Delgado-Rodríguez and Llorca, 2004). This discrepancy may artificially inflate the reported prevalence of symptoms like “brain fog” in HICs, independent of any underlying socioeconomic or biological determinant. Without harmonizing the recruitment pathways, attributing these variations to national income levels risks oversimplifying complex epidemiological data.

## 3 Trans-cultural validity of neuro-phenomenological tools

The study relies heavily on Patient-Reported Outcomes (PROs) and standardized cognitive screens such as the MoCA and PROMIS (Aaronson et al., 2002); however, the cultural and linguistic invariance of these instruments is not thoroughly addressed. The experience of “brain fog”—a term central to Western patient narratives—may not have a direct phenomenological equivalent in the linguistic contexts of the Indian or Nigerian cohorts (Henrich et al., 2010).

Furthermore, performance on cognitive screens like the MoCA is highly sensitive to educational background and formal schooling styles. If these scores were not rigorously adjusted for local educational norms, the resulting “deficits” may reflect a measurement gap rather than a biological disparity. To avoid reinforcing a “deficit model” of health in LMICs, neuroscientific research must ensure that diagnostic tools are not only translated but also culturally validated for the specific populations being studied.

## 4 Discussion

The adoption of the colloquial term “Long COVID” throughout the study further complicates the distinction between primary viral neuropathology and the secondary neuropsychiatric effects of the pandemic's socioeconomic stressors. By framing disparities primarily through the lens of the World Bank income classifications, the study may overlook inherent cultural resiliencies or alternative manifestations of neurological recovery.

In conclusion, while Jimenez et al. provide a vital global dataset, future research must control for recruitment sources and prioritize the use of culturally sensitive diagnostic frameworks. Only through such rigor can we ensure that global neuroscience accurately reflects the diverse realities of the human brain's response to the post-pandemic era, moving beyond the current status quo of clinical reporting.
